# Epidemiology of Thyroid Cancer: Incidence and Mortality in China, 2015

**DOI:** 10.3389/fonc.2020.01702

**Published:** 2020-11-10

**Authors:** Lingbin Du, Zixuan Zhao, Rongshou Zheng, Huizhang Li, Siwei Zhang, Runhua Li, Wenqiang Wei, Jie He

**Affiliations:** ^1^Department of Cancer Prevention, Institute of Cancer and Basic Medicine, Zhejiang Cancer Hospital, Chinese Academy of Sciences, Cancer Hospital of the University of Chinese Academy of Sciences, Hangzhou, China; ^2^Center for Health Policy Studies, School of Medicine, Zhejiang University, Hangzhou, China; ^3^Office for Cancer Registry, National Cancer Center, National Clinical Research Center for Cancer, Peking Union Medical College, Cancer Hospital, Chinese Academy of Medical Sciences, Beijing, China; ^4^Department of Prevention & Health Care, Institute of Cancer and Basic Medicine, Zhejiang Cancer Hospital, Chinese Academy of Sciences, Cancer Hospital of the University of Chinese Academy of Sciences, Hangzhou, China; ^5^Department of Thoracic Surgery, National Cancer Center, National Clinical Research Center for Cancer, Peking Union Medical College, Cancer Hospital, Chinese Academy of Medical Sciences, Beijing, China

**Keywords:** cancer registry, thyroid cancer, incidence, mortality, China

## Abstract

**Objective:** Using data from cancer registries to estimate thyroid cancer incidence and mortality in China, 2015.

**Methods:** Data submitted from local cancer registries were checked and evaluated according to the criteria of data quality control, a total of 368 cancer registries' data were qualified for the final analysis. Data were stratified by area (urban/rural, eastern/central/western), sex and age, combined with national population data to estimate thyroid cancer incidence and mortality in China, 2015.

**Results:** Approximately 200,700 new cases were diagnosed in 2015, accounting for 5.11% of all cancer cases. The crude incidence rate was 14.60/100,000. Age-standardized incidence rates by Chinese standard population (ASIRC) and world standard population (ASIRW) were 12.05/100,000 and 10.44/100,000, with the cumulative incidence rate (0–74 years old) of 1.00%. About 7,900 deaths of thyroid cancer were reported in 2015, accounting for 0.34% of all cancer deaths. The crude mortality rate was 0.58/100,000, age-standardized mortality rates by Chinese standard population (ASMRC) and world standard population (ASMRW) were 0.37/100,000 and 0.36/100,000. The age-standardized incidence and mortality in females were significantly higher than those in males (*P* < 0.001). The rates in urban areas were higher than those in rural areas (*P* < 0.001). The ASIRC in eastern areas was higher than that in central and western areas (*P* < 0.001), while the ASMRC in central areas was higher than that in eastern and western areas (*P* < 0.001).

**Conclusions:** The burden of thyroid cancer was heavy in China, cancer control faces the problem of the disparity between geographic areas, and the incidence and mortality rates were varied by sex and age. Targeted cancer preventive measures should be put into practice.

## Introduction

Thyroid cancer is the most common endocrine and head-and-neck malignancy globally. Since 1990s, the incidence rate of the disease has been increasing rapidly around the world excluding Africa for their limited diagnostic technology. Paes et al. reported in a research in 2010 that thyroid cancer incidence is rising at a rate that is the fastest of all malignancies (1). According to the Global Cancer Observatory (GLOBOCAN) 2018 from International Agency for Research on Cancer (IARC), about 567,200 new cases were diagnosed, and 41,100 deaths were caused by thyroid cancer in 2018 (2). The incidence rate of thyroid cancer is also increasing in China. According to the annual report of China Cancer Registry 2018, thyroid cancer has become one of the 10 major cancers threatening the health of Chinese residents (3). In some provinces (e.g., Zhejiang province), the incidence rate of thyroid cancer has dramatically risen to the top of all cancers in females (4). Some researchers have reported the incidence and mortality rates of thyroid cancer in the regional level (5–7), while the nationwide data is still lacked. As national cancer registration data were not publicly available in the same fashion as the Surveillance, Epidemiology, and End Results Program in the USA, the National Central Cancer Registry of China (NCCRC) provides a status report on the cancer incidence and mortality regularly, with the focus on geographic, sex, and age variability throughout the country (8). In this study, we extracted the latest data of thyroid cancer from the database of the NCCRC and provide a nationwide epidemiology report of thyroid cancer in 2015.

## Materials and Methods

### Data Source

**Figure 1:**
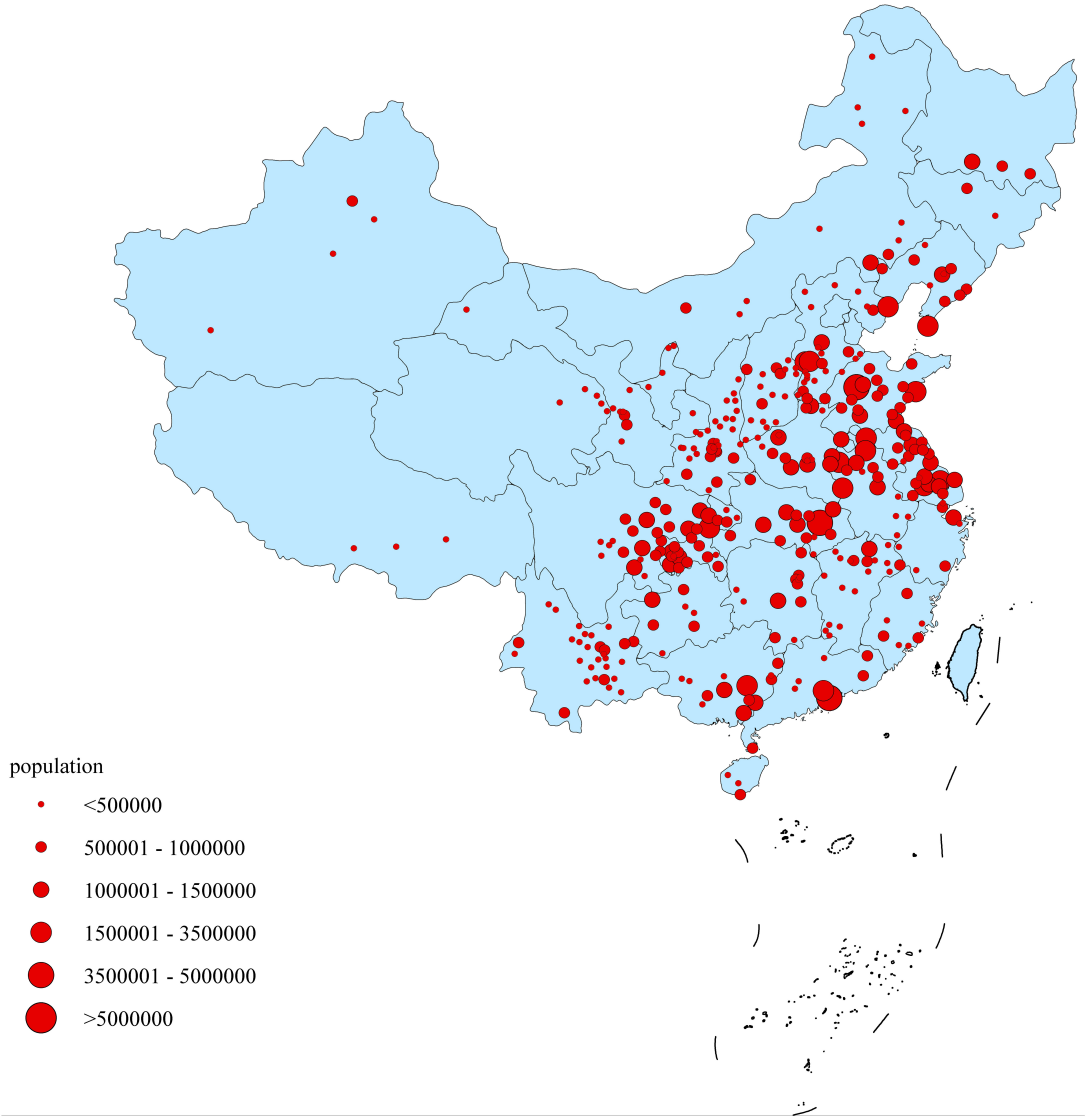
Geographical distribution of the 368 cancer registries in China, 2015.

NCCRC extracted cancer data through 501 cancer registries from 31 provinces in China, including 173 cancer registries in cities above the county level and 328 cancer registries in cities of the county level. A total of 368 cancer registries' data met the criteria of quality control and they were included in pooled data, of which 134 were located in urban areas and 234 were located in rural areas. A number of 309,553,499 populations (156,934,140 males and 152,619,359 females) were covered, accounting for 22.52% of the national population at the end of year 2015 (Figure 1). Among them, 148,804,626 (48.07%) were located in the urban areas, 160,748,873 (51.93%) located in the rural areas (9). According to International Statistical Classification of Diseases and Related Health Problems 10th Revision (ICD-10), thyroid cancer cases, which are coded as C73, were extracted for analysis. The data inclusion criteria were focused on the authenticity, stability, and comparability of data quality. The quality of data was evaluated based on the characteristics of the corresponding regions. Aside from several important indexes such as MV%, DCO%, M/I, incidence and mortality, registries with proper range of each index were taken into consideration. Specifically, MV% ranged from 66 to 85%, DCO% <15%, M/I ranged from 0.6 to 0.8 were included. Registries with poor data stability, fluctuation of incidence and mortality larger than 10% were defined as low quality and those data were excluded. To obtain the completeness of regional coverage of the country, the standard of data inclusion was not such strict to western provinces, low socio-economic areas and the minority nationality regions, allowing a 10% fluctuation on the data inclusion criteria.

### Quality Control

The “Guideline for Chinese Cancer Registration (2016)” (10) and requirement from IARC (11, 12) were used to build data inclusion criteria. Excel 2007 was used for data management, and IARCcrg Tools (Version 2.05) (13) issued by IARC/International Association of Cancer Registries' (IACR) was used for data review and evaluation. The validity, reliability, completeness, and comparability of data were evaluated based on the percentage of morphologically verified cases (MV%), the percentage of death certificate-only cases (DCO%), the mortality to incidence (M/I) ratio and the percentage of the diagnosis of unknown basis (UB%). For all kinds of cancer reported by Zheng's study, the total MV% was 69.34%, the DCO% was 2.09%, and the M/I was 0.61 (9), among which the MV%, DCO% and M/I for thyroid cancer was 93.18, 0.10, and 0.04, respectively (Table 1).

**Table 1:** Quality evaluation of thyroid cancer in China, 2015.

**Areas**	**Sex**	**M/I**	**%**		
			**MV**	**DCO**	**UB**
Both	Both	0.04	93.18	0.10	0.09
	Male	0.07	92.71	0.17	0.12
	Female	0.04	93.33	0.08	0.08
Urban areas	Both	0.04	94.70	0.08	0.05
	Male	0.05	94.51	0.11	0.06
	Female	0.03	94.76	0.07	0.05
Rural areas	Both	0.06	89.99	0.16	0.18
	Male	0.11	88.40	0.30	0.27
	Female	0.05	90.44	0.11	0.15
Eastern areas	Both	0.03	94.82	0.09	0.09
	Male	0.05	94.49	0.18	0.11
	Female	0.03	94.92	0.06	0.09
Middle areas	Both	0.07	89.97	0.09	0.00
	Male	0.10	89.84	0.11	0.00
	Female	0.06	90.01	0.08	0.00
Western areas	Both	0.08	85.22	0.29	0.32
	Male	0.12	82.94	0.25	0.49
	Female	0.07	86.01	0.30	0.26

### Statistical Analysis

The population by age, sex and area (urban/rural) in 2015 was calculated according to the 5th and 6th census data released by the National Bureau of statistics, combined with the population of China from 2000 to 2015, the change of urban-rural ratio, and the age composition of the Chinese population.

Crude incidence and mortality rates of thyroid cancer were calculated in each stratum by age group (0–, 1–4, 5–84 by 5 years and 85+ years old), sex and area (urban/rural). The number of new cases and deaths of thyroid cancer were estimated in China, 2015. Age-standardized rates by Chinese and world standard population were calculated using the population of China in 2000 and Segi's population structure, respectively (14). Urban and rural areas were classified by the regulation of National Bureau of Statistics of the People's Republic of China, and the incidence (death) rate, standardized incidence (death) rate, composition ratio, cumulative incidence (death) rate (0–74 years old) and truncated rate (35–64 years old) were calculated, respectively. SAS software (Version 9.4; SAS Institute Inc., Cary, USA) was used for statistical analysis. Formulae used are as follows:

(1) $$\begin{array}{l} \text{Incidence }\left(\text{mortality}\right)\text{ rate per}100,000=\frac{\text{new cases }\left(\text{new cancer death}\right)\text{ occurring during a given period }}{population\;at\;risk\;at\;the\;same\;period{\;}^{*}100,000\;} \end{array}$$

(2) $$\begin{array}{l} \text{Age}-\text{specific incidence }\left(\text{mortality}\right)\text{ rate per}100,000=\frac{\text{cases in a specific age group}}{\text{population in the age group}{\;}^{*}100,000\;} \end{array}$$

(3) $$\begin{array}{l} \text{ASR per }100,000=\frac{\sum \left(\text{standard population in corresponding age grou}{\text{p}}^{*}\text{age}-\text{specific rate}\right)}{\sum standard\;population} \end{array}$$

(4) $$\begin{array}{l} \text{Cumulative rate}\left(\%\right)={\left(\sum \left(age-specific\;rate^{*}width\;of\;the\;age\;group\right)\right)}^{*}100 \end{array}$$

(5) $$\text{Truncated incidence (mortality) rate per}100,000=\frac{\sum \text{trancated rate in a specific age group*}standard\;population\;of\;the\;age\;group\text{)}}{\sum \text{standard population}}$$

## Results

### Incidence of Thyroid Cancer

In 2015, the estimated new cases diagnosed with thyroid cancer were 200,700 in China, accounting for 5.11% of all new cancer cases, with the incidence rate ranked 7th in all kinds of cancer. The crude incidence rate was 14.60/100,000, the age-standardized incidence rate by Chinese standard population (ASIRC) and world standard population (ASIRW) were 12.05/100,000 and 10.44/100,000, and the cumulative rate (0–74 years old) was 1.00%. The incidence rate of thyroid cancer showed a gender variance in China, the ASIRC in males and females were 6.01 and 18.29/100,000, ranking 11st and 4th separately with the sex ratio of 3.04 (Table 2). In urban and rural areas, the estimated new cases were 151,000 and 49,700 cases, of which incidence rate in urban areas was 19.59/100,000 (9.80/100,000 in males, 29.79/100,000 in females), in rural areas was 8.24/100,000 (3.52/100,000 for males, 13.25/100,000 for females). The ASIRC in urban and rural areas were 15.60 and 7.06/100,000 with the ASIRC ratio of 2.21.

**Table 2:** Estimated new cases and incidence rate of thyroid cancer in China, 2015.

**Areas**	**Sex**	**New cases (×10^**4**^)**	**Crude incidence (1/10^**5**^)**	**Ratio (%)**	**ASIRC (1/10^5^)**	**ASIRW (1/10^5^)**	**Cumulative rate 0–74 (%)**	**Truncated rate 35–64 (1/10^5^)**	**Rank**	***P*-value**
All areas	Both	20.07	14.60	5.11	12.05	10.44	1.00	23.30	7	
	Male	4.94	7.03	2.30	6.01	5.12	0.49	10.90	11	<0.001
	Female	15.12	22.56	8.51	18.29	15.94	1.53	36.05	4	
Urban areas	Both	15.10	19.59	6.42	15.60^a^	13.47	1.28	29.33	6	
	Male	3.85	9.80	3.06	8.09	6.85	0.64	14.21	8	<0.001
	Female	11.24	29.79	10.29	23.38	20.34	1.94	45.04	3	
Rural areas	Both	4.97	8.24	3.15	7.06^a^	6.24	0.61	14.07	7	
	Male	1.09	3.52	1.22	3.11	2.73	0.27	5.78	15	<0.001
	Female	3.87	13.25	5.66	11.13	9.85	0.95	22.48	8	
Eastern areas	Both	11.99	23.26	7.36	19.23^b^	16.45	1.56	36.46	6	
	Male	2.95	11.29	3.41	9.76	8.18	0.76	17.35	7	<0.001
	Female	9.04	35.57	11.83	28.80	24.83	2.36	55.71	3	
Central areas	Both	5.35	11.60	4.09	9.32^b^	8.21	0.80	18.93	7	
	Male	1.26	5.31	1.75	4.41	3.84	0.37	8.51	13	<0.001
	Female	4.09	18.28	6.99	14.43	12.75	1.24	29.72	6	
Western areas	Both	2.72	6.85	2.75	5.72^b^	5.03	0.49	10.82	8	
	Male	0.72	3.56	1.29	3.05	2.64	0.26	5.37	15	<0.001
	Female	1.99	10.36	4.66	8.55	7.56	0.73	16.55	7	

*ASIRC, age-standardized incidence rate adjusted by Chinese standard population; ASIRW, age-standardized incidence rate adjusted by world population*.

a *ASIRC between urban and rural areas, P < 0.001*.

b *ASIRC between eastern, central and western areas, P < 0.001*.

The new cases of thyroid cancer diagnosed were about 119,900, 53,500, and 27,200 cases in the eastern, central, and western areas. The incidence rate in the eastern areas was 23.26/100,000 (11.29/100,000 in males, 35.57/100,000 in females), much higher than 11.60/100,000 in the central areas (5.31/100,000 in males, 18.28/100,000 in females) and 6.85/100,000 in the western areas (3.56/100,000 in males, 10.36/100,000 in females). Eastern areas had the highest ASIRC (19.23/100,000), followed by central areas (9.32/100,000) and western areas (5.72/100,000). After adjusting age composition, the gap of incidence rate in different areas narrowed, but still remained the similar trend. In any areas, incidence in females was higher than that in males (*P* < 0.001) (Table 2).

### Age-Specific Incidence Rate of Thyroid Cancer

**Figure 2:**
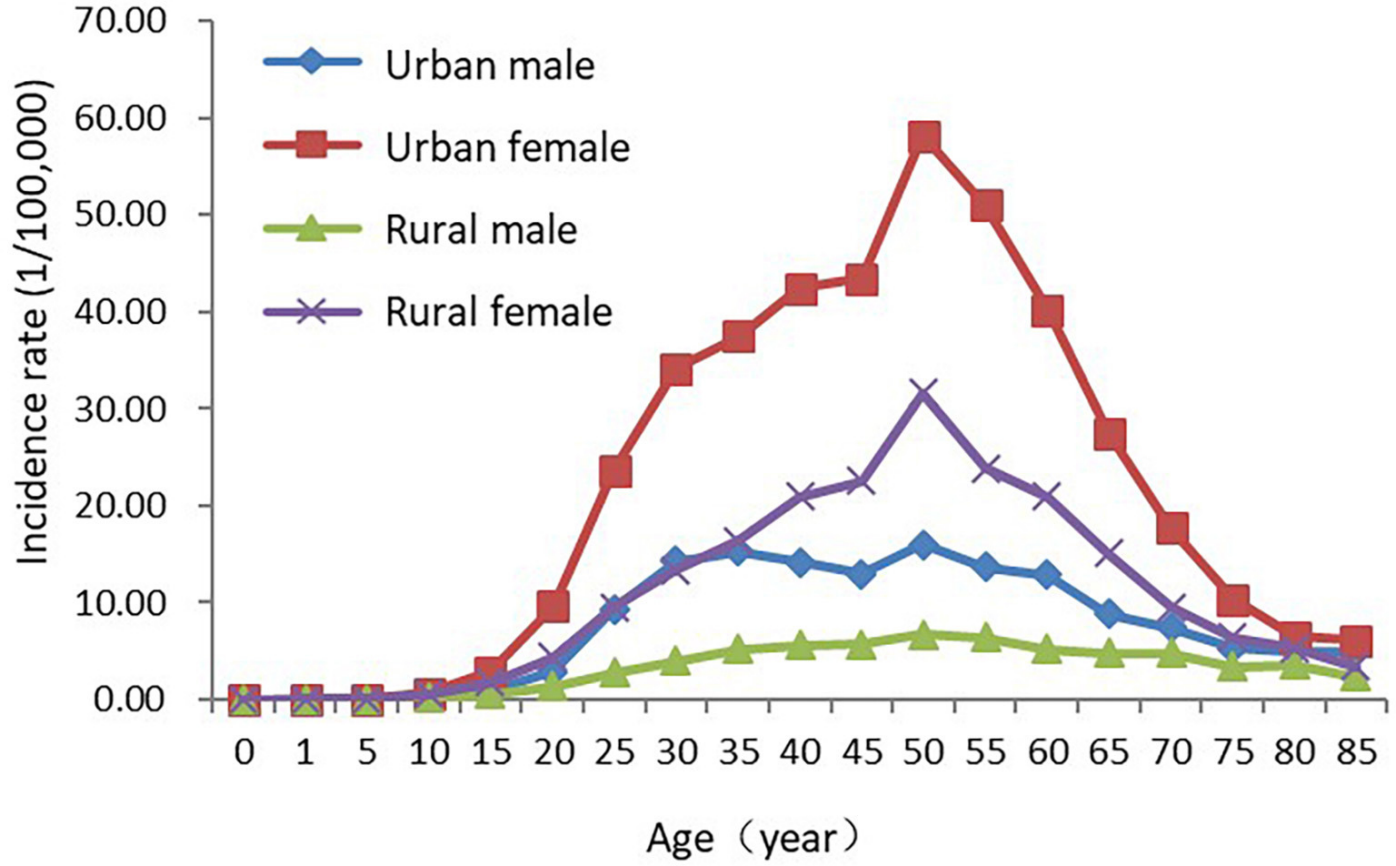
Age-specific thyroid cancer incidence rate in China, 2015.

Age-specific incidence rates increased with the growth of age. It remained relatively low before 15 years old and increased rapidly afterwards, reaching a peak in the age group of 50–54 years old. It began to decline after 55 years old, and dropped to 4.60/100,000 at 85+ years old. In the majority of age groups, incidence rates in females were higher than those in males with the sex ratio of about 3.00 at 15–70 years old. Age-specific incidence rates reached the peaks at the 50–54 years old both in males (12.40/100,000) and females (47.46/100,000). The incidence rates of urban and rural areas both reaching the peaks in the age group of 50–54 years old, while in most of age groups, age-specific incidence rates in urban areas were higher than those in rural areas (Table 3, Figure 2).

**Table 3:** Age-specific incidence rate of thyroid cancer in China, 2015 (1/10^5^).

**Age group**	**All areas**			**Urban areas**			**Rural areas**			***P*-value^**a**^**	***P*-value^**b**^**
	**Both**	**Male^a^**	**Female^a^**	**Both^b^**	**Male**	**Female**	**Both^b^**	**Male**	**Female**		
Total	14.60	7.03	22.56	19.59	9.80	29.79	8.24	3.52	13.25	<0.001	<0.001
0–	0.00	0.00	0.00	0.00	0.00	0.00	0.00	0.00	0.00	–	–
1–	0.01	0.00	0.01	0.00	0.00	0.00	0.01	0.00	0.03	0.284	0.376
5–	0.05	0.03	0.07	0.08	0.06	0.10	0.02	0.00	0.05	0.236	0.055
10–	0.35	0.15	0.58	0.37	0.13	0.64	0.33	0.18	0.52	<0.001	0.65
15–	1.44	0.68	2.34	1.78	0.83	2.88	1.09	0.53	1.76	<0.001	<0.001
20–	4.36	2.13	6.91	6.06	2.92	9.61	2.81	1.41	4.42	<0.001	<0.001
25–	10.78	5.84	16.11	16.22	9.35	23.67	6.02	2.75	9.52	<0.001	<0.001
30–	18.03	10.07	26.16	24.27	14.26	34.15	8.50	3.95	13.41	<0.001	<0.001
35–	20.57	11.52	29.70	26.37	15.28	37.49	10.77	5.22	16.43	<0.001	<0.001
40–	22.32	10.83	34.12	28.18	14.19	42.47	13.13	5.58	20.94	<0.001	<0.001
45–	22.12	9.98	34.72	27.79	12.93	43.49	14.07	5.70	22.54	<0.001	<0.001
50–	29.58	12.40	47.46	36.36	15.98	58.15	19.20	6.73	31.64	<0.001	<0.001
55–	25.30	10.88	40.38	31.83	13.67	51.02	15.04	6.42	23.89	<0.001	<0.001
60–	20.75	9.62	31.90	26.51	12.89	40.17	13.08	5.25	20.91	<0.001	<0.001
65–	14.51	7.01	21.98	18.18	8.79	27.46	9.92	4.79	15.06	<0.001	<0.001
70–	10.27	6.23	14.11	12.75	7.45	17.67	7.14	4.75	9.50	<0.001	<0.001
75–	6.74	4.52	8.73	7.99	5.33	10.32	4.98	3.42	6.43	<0.001	<0.001
80–	5.31	4.39	6.05	5.83	4.96	6.56	4.55	3.55	5.33	0.012	0.045
85+	4.60	3.98	5.00	5.66	4.96	6.15	3.04	2.36	3.43	0.166	<0.001

### Mortality Rate of Thyroid Cancer

In 2015, thyroid cancer was 22nd leading cause of cancer-related deaths in China, ranking 19th in males and 20th in females. It is estimated 7,900 thyroid cancer deaths in 2015, accounting for 0.34% of all cancer deaths. The mortality rate of thyroid cancer was higher in females than that in males, the sex ratio of age-standardized mortality rate by Chinese standard population (ASMRC) was 1.59 (ASMRC in males was 0.29/100,000 and in females was 0.46/100,000) (Table 4).

**Table 4:** Estimated deaths and mortality rate of thyroid cancer in China, 2015.

**Areas**	**Sex**	**Deaths (×10^4^)**	**Crude mortality (1/10^5^)**	**Ratio (%)**	**ASMRC (1/10^5^)**	**ASMRW (1/10^5^)**	**Cumulative rate 0–74 (%)**	**Truncated rate 35–64 (1/10^5^)**	**Rank**	***P*-value**
All areas	Both	0.79	0.58	0.34	0.37	0.36	0.04	0.53	22	
	Male	0.30	0.43	0.20	0.29	0.28	0.03	0.43	19	<0.001
	Female	0.49	0.73	0.57	0.46	0.44	0.05	0.64	20	
Urban areas	Both	0.49	0.64	0.37	0.40^a^	0.38	0.04	0.56	22	
	Male	0.18	0.48	0.22	0.31	0.30	0.03	0.44	19	<0.001
	Female	0.30	0.81	0.62	0.48	0.46	0.05	0.68	20	
Rural areas	Both	0.29	0.50	0.30	0.34^a^	0.33	0.04	0.50	22	
	Male	0.11	0.37	0.18	0.26	0.26	0.03	0.41	19	<0.001
	Female	0.18	0.63	0.51	0.41	0.40	0.04	0.59	20	
Eastern areas	Both	0.29	0.58	0.32	0.34^b^	0.33	0.04	0.45	23	
	Male	0.11	0.44	0.20	0.28	0.27	0.03	0.38	19	<0.001
	Female	0.18	0.71	0.52	0.40	0.39	0.04	0.53	20	
Central areas	Both	0.31	0.69	0.40	0.47^b^	0.44	0.05	0.71	22	
	Male	0.11	0.48	0.22	0.33	0.32	0.03	0.56	19	<0.001
	Female	0.20	0.91	0.69	0.60	0.56	0.06	0.86	20	
Western areas	Both	0.17	0.45	0.29	0.30^b^	0.30	0.03	0.43	22	
	Male	0.07	0.35	0.18	0.25	0.25	0.03	0.34	19	<0.001
	Female	0.10	0.55	0.49	0.36	0.36	0.04	0.53	20	

*ASMRC, age-standardized mortality rate adjusted by Chinese standard population; ASMRW, age-standardized mortality rate adjusted by world standard population*.

a *ASMRC between urban and rural areas, P < 0.001*.

b *ASMRC between eastern, central and western areas, P < 0.001*.

In urban and rural areas, the estimated thyroid cancer deaths were 4,900 and 2,900, respectively. The mortality rate of thyroid cancer in urban areas was 0.64/100,000 (0.48/100,000 in males, 0.81/100,000 in females), which was higher than that in rural areas (0.37/100,000 in males, 0.63/100,000 in females). The ASMRC in urban and rural areas were 0.40/100,000 and 0.34/100,000 with the ASMRC ratio of 1.18.

The new deaths of thyroid cancer were about 2,900, 3,100 and 1,700 cases in the eastern, central and western areas. Among them, the mortality rate in the central areas was 0.69/100,000 (0.48/100,000 in males, 0.91/100,000 in females), slightly higher than 0.58/100,000 in the eastern areas (0.44/100,000 in males, 0.71/100,000 in females) and 0.45/100,000 in the western areas (0.35/100,000 in males, 0.55/100,000 in females). Central areas had the highest ASMRC (0.47/100,000), followed by eastern areas (0.34/100,000) and western areas (0.30/100,000). The gap of mortality rates in different areas significantly narrowed after adjusting age composition, but still remained the similar trend. In any areas, the mortality rates of thyroid cancer in females were also higher than that in males as incidence rates showed above (Table 4).

### Age-Specific Mortality Rate of Thyroid Cancer

**Figure 3:**
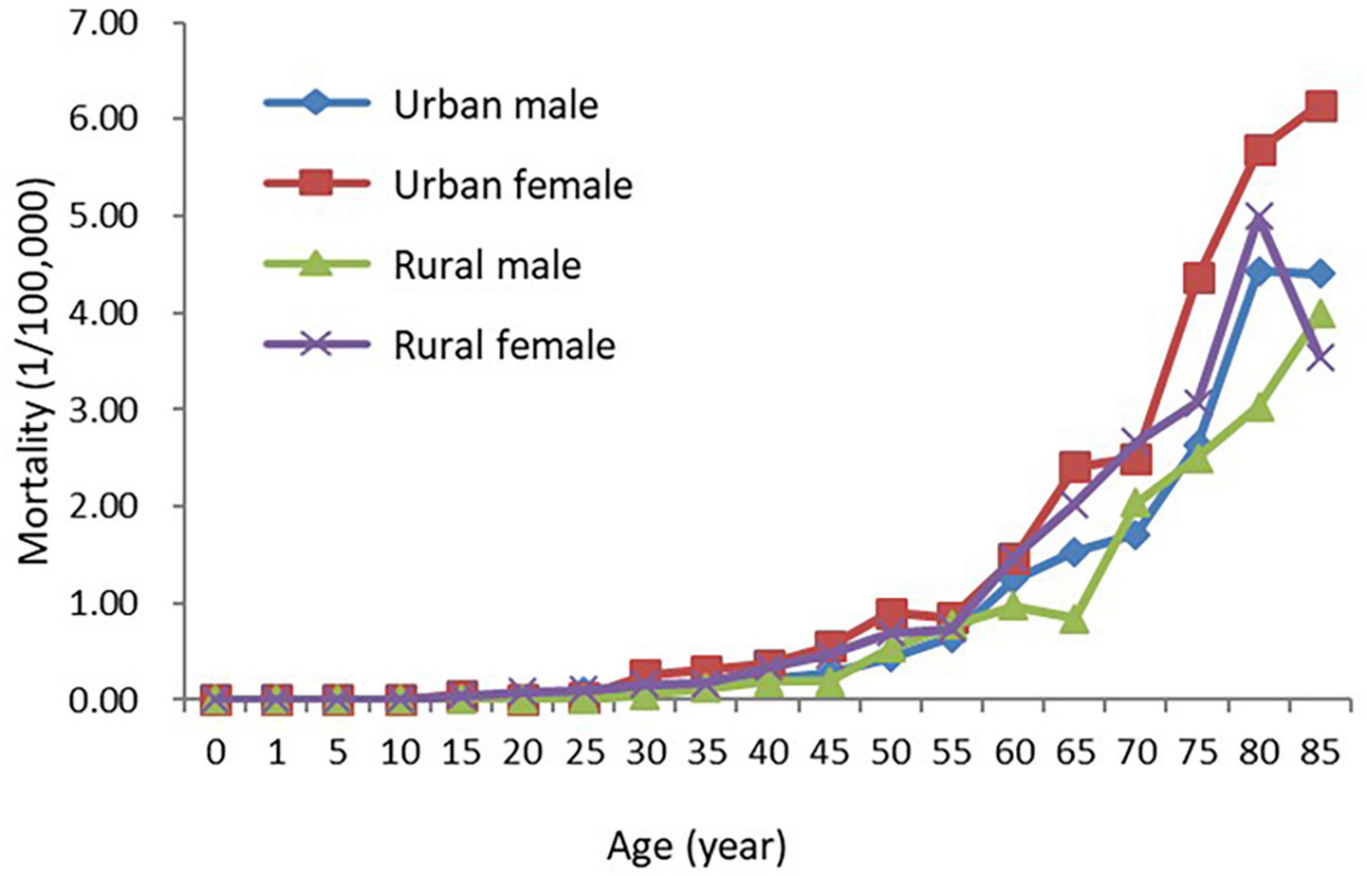
Age-specific thyroid cancer mortality rate in China, 2015.

Age-specific mortality rates increased with the growth of age, reaching a peak in the age group of 85+ years old. In different genders, the mortality rate of thyroid cancer also increased with the growth of age. The mortality rates reached their peaks in the age group of 85+ years old in males (4.25/100,000) and 80–84 years old in females (5.40/100,000). In each age group, however, differences in mortality rates in different genders were not statistically significant, so as in different areas In urban and rural areas, mortality rates reached their peaks in the age group of 85+ years old in urban areas (5.44/100,000) and 80–84 years old in rural areas (4.14/100,000) (Table 5, Figure 3).

**Table 5:** Age-specific mortality of thyroid cancer in China, 2015 (1/10^5^).

**Age group**	**All areas**			**Urban areas**			**Rural areas**			***P*-value^a^**	***P*-value^b^**
	**Both**	**Male^a^**	**Female^a^**	**Both^b^**	**Male**	**Female**	**Both^b^**	**Male**	**Female**		
Total	0.58	0.43	0.73	0.64	0.48	0.81	0.50	0.37	0.63	<0.001	<0.001
0–	0.00	0.00	0.00	0.00	0.00	0.00	0.00	0.00	0.00	–	–
1–	0.00	0.00	0.00	0.00	0.00	0.00	0.00	0.00	0.00	–	**–**
5–	0.00	0.00	0.00	0.00	0.00	0.00	0.00	0.00	0.00	–	–
10–	0.00	0.00	0.00	0.00	0.00	0.00	0.00	0.00	0.00	–	–
15–	0.03	0.01	0.05	0.04	0.03	0.06	0.02	0.00	0.05	0.156	0.414
20–	0.03	0.01	0.05	0.01	0.00	0.02	0.05	0.02	0.09	0.053	0.059
25–	0.05	0.04	0.07	0.06	0.08	0.03	0.05	0.00	0.11	0.264	0.796
30–	0.14	0.06	0.22	0.16	0.07	0.26	0.11	0.05	0.16	0.001	0.289
35–	0.21	0.15	0.26	0.24	0.17	0.31	0.15	0.12	0.18	0.052	0.109
40–	0.28	0.20	0.36	0.29	0.21	0.37	0.26	0.19	0.34	0.013	0.651
45–	0.37	0.24	0.52	0.41	0.27	0.55	0.33	0.19	0.46	<0.001	0.253
50–	0.64	0.47	0.81	0.66	0.44	0.90	0.61	0.54	0.69	0.001	0.574
55–	0.75	0.69	0.81	0.75	0.64	0.86	0.76	0.78	0.74	0.311	0.936
60–	1.30	1.12	1.47	1.35	1.24	1.46	1.22	0.97	1.47	0.034	0.402
65–	1.73	1.23	2.24	1.98	1.54	2.41	1.43	0.84	2.02	<0.001	0.017
70–	2.22	1.86	2.57	2.12	1.71	2.50	2.35	2.03	2.67	0.019	0.457
75–	3.24	2.58	3.84	3.56	2.64	4.37	2.80	2.50	3.08	0.002	0.073
80–	4.72	3.87	5.40	5.12	4.43	5.68	4.14	3.03	5.00	0.009	0.129
85+	4.73	4.25	5.04	5.44	4.41	6.15	3.70	3.99	3.54	0.256	0.028

### Histologic Subtypes Distribution of Thyroid Cancer

**Figure 4:**
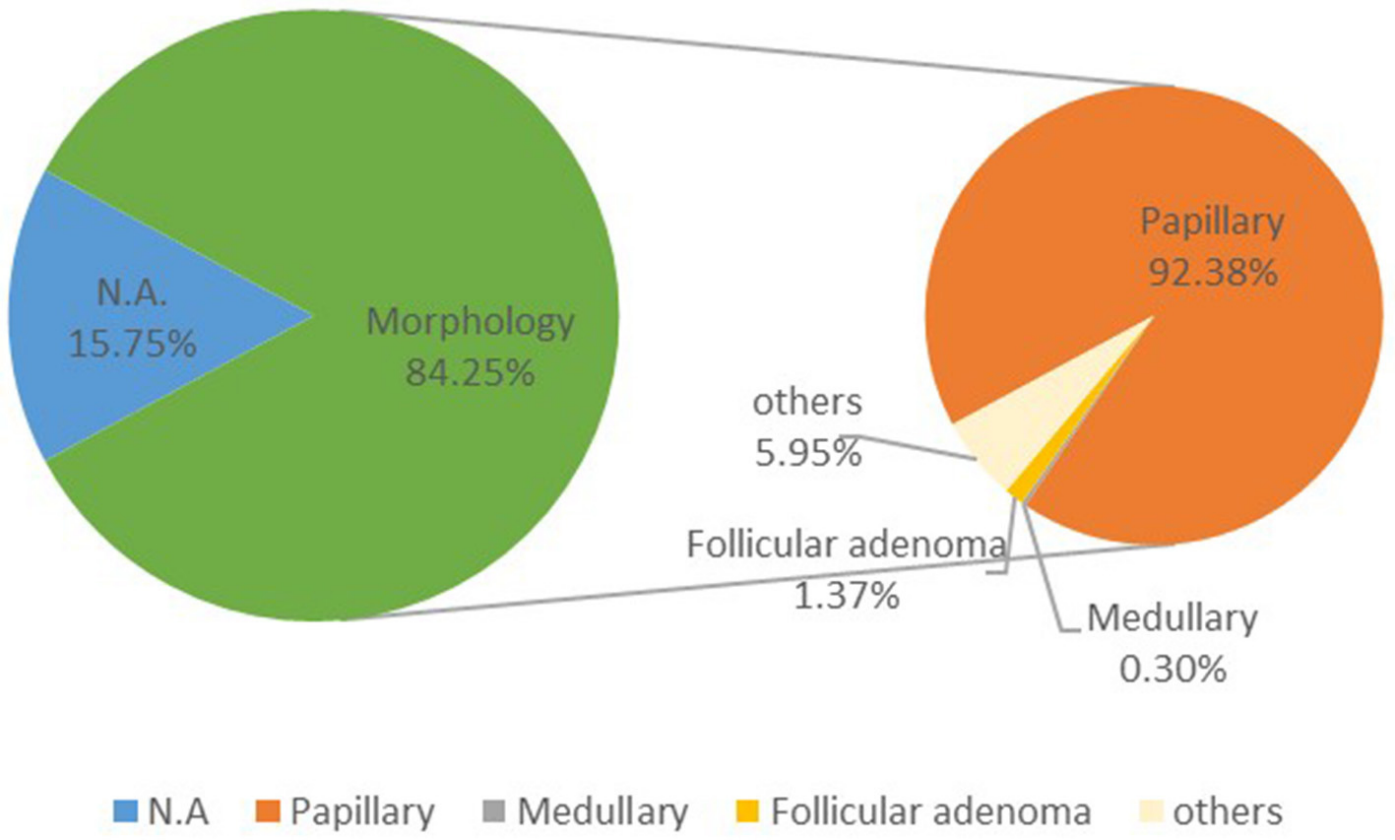
Histologic subtypes distribution of thyroid cancer in China, 2015.

Thyroid cancer contains papillary thyroid carcinoma (PTC), medullary thyroid carcinoma (MTC), follicular thyroid carcinoma (FTC), and poorly differentiated thyroid cancer (PDTC), etc. In 2015, about 84.25% cases of thyroid cancer had morphological verification. The most common type of thyroid cancer is PTC (92.38%), whereas FTC and MTC comprise only ~1.37 and 0.30% of thyroid cancer, and other less common variants encompass the small subset of remaining cases (Figure 4).

## Discussion

According to GLOBOCAN 2018, it was estimated that there were 567,200 new diagnosed cases of thyroid cancer in 2018 around the world. The ASIRW and cumulative rate in 0–74 years old of thyroid cancer were 6.7/100,000 and 0.68%. The number of newly diagnosed cases in China is 194,200, accounting for 34.23% of the overall new cases worldwide. The distribution of thyroid cancer showed geographically variance globally, 59.98% occurred in Asia, 13.82% occurred in Europe, 12.44% occurred in North America, and Africa accounted for only 3.22%. The highest estimated incidence rate was found in Korea, with the incidence rate of 60.7/100,000 (2).

This study estimated thyroid cancer burden in China in 2015 using the data of 368 local cancer registries that met the inclusion criteria, accounting for ~22.52% of the Chinese population. The calculation of incidence and mortality rates were based on the census data released by the National Bureau of Statistics, which was more accurate than the data from GLOBOCAN2018 and could better describe the current status of cancer epidemics in China. The estimated number of thyroid cancer cases newly diagnosed in China in 2015 was about 200,700, ranking 7th among new cases of malignant tumors and had become one of the top 10 cancers threatening health of Chinese residents. This increase is most probably linked to diagnostic progresses and more neck scans in China. Since 2010, some provinces in China start to incorporate the neck routine scan into the regular examination for urban employees, as well as the improvement of public awareness toward cancer prevention have led to the “high incidence” of thyroid cancer in recent years. In 2015, the incidence rates of thyroid cancer in China (ASIRW: 10.44/100,000) were higher than the world average (ASIRW: 6.7/100,000) and ranked 19th worldwide. The ASIRW in 2015 estimated by our team was higher than that estimated by GLOBCAN2018(ASIRW: 10.1/100,000) (15), which further confirmed the strength of our study, for we have a much larger data source than any other studies also investigating the incidence and mortality of thyroid cancer in China. Thyroid cancer had relatively low mortality rates in the past few decades, and it was the 22nd leading cause of cancer in China. The age-standardized mortality rate standard population (ASMRW) in China was 0.36/100,000, lower than the world average of 0.42/100,000 in 2015(3). In our study, the incidence and mortality rates of thyroid cancer varied by sex, age groups, and areas. The results were consistent with the findings of incidence rates are usually higher in higher Human Development Index (HDI) vs. lower HDI settings, while mortality rates, in contrast, are rather similar (15). The explanation for the differences in incidence among different sex and areas are controversial, and several causes have been hypothesized; increased diagnostic intensity as well as “over-diagnosis” in females (16), and tumourigenesis in thyroid tissues is influenced by endocrine function and hormones, particularly oestrogen (17). In addition to, the environmental exposures in different HDI settings may be contributing to the observed variance in different areas (18). Priority should be placed on research to identify modifiable risk factors for females and populations in high HDI areas (e.g., urban areas, eastern areas), as well as improvement of public awareness for cancer prevention in males and populations in low HDI areas (e.g., rural areas, western areas). It is of note that the mortality rates in central areas (0.47 per 100,000) were significantly higher than those observed in other areas [e.g., in eastern areas [0.34 per 100,000], despite central areas [9.32 per 100,000] had a substantially lower incidence than eastern areas [19.23 per 100,000]]. As previous studies only focused on the differences between urban and rural areas when measuring regional variations (19, 20), the trend of mortality rates in central areas cannot be obtained. The results prompt the need of prudent consideration for greater focus on the cause of the relatively high mortality rate in central areas in China. The mortality rates of thyroid cancer increased with age like other most common cancer types. And they increased reaching a peak in the age group of 80–84 years old (in males and urban areas) and a peak in the 85+ years old (in females and rural areas).

Thyroid cancer incidence has been rising with the fastest speed over the last decades worldwide (21, 22), the ASIRC increased dramatically by 4.73 times between 2003 and 2012, from 2.40/100,000 to 13.75/100,000 in China (23). Compared with the data in recent years, the incidence and mortality rate of thyroid cancer in China also showed an increasing trend, with the incidence rising from 8.82/100,000 in 2013 to 12.05/100,000 in 2015. The mortality rate slightly rose from 0.33/100,000 in 2013 to 0.37/ 100,000 in 2015. The same trend was observed in different areas and genders (20).

Based on our past work, we observed a rapid increase in the proportions of PTC in 12,508 patients with thyroid carcinoma identified in the Zhejiang Cancer Hospital from 1972 to 2014 while the proportions of MTC, FTC, and PDTC decreased over the decades. In the PTC cases, the proportion of patients with maximum tumor diameter (MTD) <1 cm dramatically and significantly increased from 0 in 1972–1985 to 32.1% in 2000–2014 (24) A higher PTC proportion was also observed in 2015 (PTC: 92.38%) compared to 2010 (PTC: 86%) (16), consistent with our previous finding. In 2015, it was estimated that there were 200,700 thyroid cancer incident cases and 7,900 deaths with the M/I of 0.04 in China. In the United States, ~62,450 new cases of thyroid cancer were predicted to be diagnosed in 2015 by the American Thyroid Association (ATA), with the predicted deaths number of 1,950. The M/I were 0.04 and 0.03 for China and the United States (25), which demonstrates thyroid cancer is usually highly treatable and is often cured with surgery. Based on the report from National Cancer Center in China, the 5-year survival for thyroid cancer increased significantly from 67.5% in 2003–05 to 84.3% in 2012–15 (26). However, the 5-year survival in China was still much lower than the United States (98.7%) (27). Similar or larger survival gaps were also found between China and other developed countries (28). Thus, much remains to be done to reduce the survival gap between China and developed countries.

There are debates over the increasing incidence of thyroid cancer internationally, doubting if the mass application of imaging technology that makes the thyroid cancer over diagnosed (29). An International Age-Period-Cohort Analysis reported that global long-term declines in thyroid cancer mortality have been accompanied by downward trends in both period and cohort effects, indicating lack of evidence of a possible major contribution of “real” risk factors in thyroid cancer mortality, and indirectly confirming the main role of over diagnosis in the epidemic of thyroid cancer incidence (30).

However, another very influential factor, the tumor sizes at the time of diagnosis, should have been considered as well but that information was not available for all analyzed cases. The investigation of tumor sizes at diagnosis could partly answer whether the recent rapid growth of thyroid cancer should be attributed to over-diagnosis. If large tumors were observed to have similar increasing trend like small tumors, then risk factors other than diagnostic progresses may have also been responsible for the increase in the incidence of thyroid cancer.

## Conclusions

Although thyroid cancer was found burdened with the high incidence, the mortality rate remained relatively low in China in 2015. The incidence and mortality rates of thyroid cancer varied by sex, age groups, and areas. Notably, mortality ASRs seem to now be high in central areas of china compared to others. This calls for further investigation of related risk factors to avoid harm in a growing number of populations. It remains utmost important to put targeted prevention and treatment programs into practice to curb the rapid growth of thyroid cancer.

## Data Availability Statement

The raw data supporting the conclusions of this article will be made available by the authors, without undue reservation.

## Author Contributions

WW and JH initiated, planned, and designed the study. LD had full access to all the data in the study. SZ conducted the data acquisition, management, and analysis. HL provided the statistical input for the data analysis. LD and ZZ drafted the manuscript. All authors interpreted the study results and critically revised the manuscript.

## Conflict of Interest

The authors declare that the research was conducted in the absence of any commercial or financial relationships that could be construed as a potential conflict of interest.

## References

[1] Paes JE Hua K Nagy R Kloos RT Jarjoura D Ringel MD. The relationship between body mass index and thyroid cancer pathology features and outcomes: a clinicopathological cohort study. J Clin Endocrinol Metab. (2010) 95:4244–50. 10.1210/jc.2010-0440 20519347 PMC2936072

[2] Freddie B Jacques F Isabelle S Rebecca LS Lindsey AT Ahmedin J Global cancer statistics 2018: GLOBOCAN estimates of incidence and mortality worldwide for 36 cancers in 185 countries. CA Cancer J Clin. (2018) 68:394–424. 10.3322/caac.21492 30207593

[3] He J Wei WQ Zhang SW Zheng RS Ma F Wang N 2018 China Cancer Registry Annual Report. Beijing: People's Medical Publishing House (2019).

[4] Yuan R Ge M Du L Li HZ Yu M Zhu C 2016 Zhejiang Cancer Registration Annual Report. Beijing: Tsinghua University Publishing House (2019).

[5] Luo T Meng D Zhang H Li FF Ning F Wang SJ Incidence and mortality of thyroid cancer in Qingdao from 2010 to 2017. Chin J Cancer Prev Treat. (2019) 26:1231–6. 10.16073/j.cnki.cjcpt.2019.17.01

[6] Ren Y Liu Q Ge M Li H Liu B Zhang Y Analysis of incidence and mortality of thyroid cancer in tumor registration areas of Zhejiang Province from 2010 to 2014. Chin J Prev Med. (2019) 1062–5. 10.19401/j.cnki.1007-3639.2019.02.001 31607057

[7] Bao P Wu C Zhang M Peng P Wang CF Gong YM Epidemiological characteristics of malignant tumors in Shanghai in 2015. Chin J Cancer. (2019) 29:81–99. 10.3760/cma.j.issn.0253-9624.2019.10.021

[8] Wei WQ Zeng HM Zheng RS Zhang SW Lan A He J. Cancer registration in China and its role in cancer prevention and control. Lancet Oncol. (2020) 21:e342–9. 10.1016/S1470-2045(20)30073-5 32615118

[9] Zheng RS Sun KX Zhang SW Zeng HM Zou X Chen R. Report of cancer epidemiology in China, 2015. Zhonghua Zhong Liu Za Zhi. (2019) 41:19–28. 10.3760/cma.j.issn.0253-3766.2019.01.005 30678413

[10] National Cancer Center Guideline for Chinese Cancer Regisitration. Beijing: People's Medical Publishing House (2016). p. 59–75.

[11] Forman D Bray F Brewster DH Gombe MC Kohler B Piñeros M Cancer Incidence in Five Continents. Vol X Lyon: IARC Scientific Publications (2014). p. 89–97.

[12] Bray F Parkin DM. Evaluation of data quality in the cancer registry: principles and methods. Part I: comparability, validity and timeliness. Eur J Cancer. (2009) 45:747–55. 10.1016/j.ejca.2008.11.032 19117750

[13] Ferlay J Burkhard C Whelan S Parkin DM Check and Conversion Programs for Cancer Registries. IARC/IACR Tools for Cancer Registries. Available online at: http://www.iacr.com.fr/images/doc/TechRep42.pdf

[14] Bray F Guilloux A Sankila R Parkin DM. Practical implications of imposing a new world standard population. Cancer Cause Control. (2002) 13:175–82. 10.1023/A:1014344519276 11936824

[15] Ferlay J Ervik M Lam F Colombet M Mery L Piñeros M Global Cancer Observatory: Cancer Today. Lyon, France: International Agency for Research on Cancer (2018). Available online at: https://gco.iarc.fr/today (accessed July 4, 2020).

[16] Yang L Wang N Advances in epidemiology of thyroid cancer. Chin J Prev Med. (2014) 48:744–8. 10.3760/cma.j.issn.0253-9624.2014.08.021

[17] Zeng Q Chen GG Vlantis AC van Hasselt CA. Oestrogen mediates the growth of human thyroid carcinoma cells via an oestrogen receptor-ERK pathway. Cell Prolif. (2007) 40:921–35. 10.1111/j.1365-2184.2007.00471.x 18021179 PMC6495898

[18] Seib CD Sosa JA. Evolving understanding of the epidemiology of thyroid cance. Endocrinol Metab Clin North Am. (2018) 48:23–35. 10.1016/j.ecl.2018.10.002 30717905

[19] Yang L Zheng R Wang N Zhang SW Chen WQ. Incidence and mortality of thyroid cancer in China in 2010. Chin J Prev Med. (2014) 48:663–8. 10.3760/cma.j.issn.0253-9624.2014.08.003 25388459

[20] Yang L Zheng RS Wang N Zeng HM Yuan YN Zhang SW. Incidence and mortality of thyroid cancer in China in 2013. Chin J Oncol. (2017) 39:862–7. 10.3760/cma.j.issn.0253-3766.2017.11.010 29151294

[21] Leenhardt L Grosclaude P Chérié-Challine L Thyroid Cancer Committee. Increased incidence of thyroid carcinoma in France: a true epidemic or thyroid nodule management effects? Report from the French Thyroid Cancer Committee. Thyroid. (2004) 14:1056–60. 10.1089/thy.2004.14.1056 15650358

[22] Wiltshire JJ Drake TM Uttley L Sabapathy PB. Systematic review of trends in the incidence rates of thyroid cancer. Thyroid. (2016) 26:1541–52. 10.1089/thy.2016.0100 27571228

[23] Du L Li R Ge M Wang YQ Li HZ Chen WQ. Incidence and mortality of thyroid cancer in China, 2008-2012. Chin J Cancer Res. (2019) 31:144–51. 10.21147/j.issn.1000-9604.2019.01.09 30996572 PMC6433579

[24] Du L Wang Y Sun X Li HZ Geng XW Ge MH. Thyroid cancer: trends in incidence, mortality and clinical-pathological patterns in Zhejiang Province, Southeast China. BMC Cancer. (2018) 18:291. 10.1186/s12885-018-4081-7 29544469 PMC5856225

[25] Haugen BR Alexander EK Bible KC Gerard MD Susan JM Yuri EN. 2015 American Thyroid Association Management Guidelines for Adult Patients with Thyroid Nodules and Differentiated Thyroid Cancer: The American Thyroid Association Guidelines Task Force on Thyroid Nodules and Differentiated Thyroid Cancer. Thyroid. (2016) 26:1–133. 10.1089/thy.2015.0020 26462967 PMC4739132

[26] Zeng H Chen W Zheng R Zhang SW John SJ Zou XN. Changing cancer survival in China during 2003-15: a pooled analysis of 17 population-based cancer registries. Lancet Glob Health. (2018) 6:e555–67. 10.1016/S2214-109X(18)30127-X 29653628

[27] National Cancer Institute Surveillance Epidemiology and End Results (SEER) Program. Cancer Query System: SEER Survival Statistics. Available online at: https://seer.cancer.gov/statfacts/html/all.html

[28] La Vecchia C Malvezzi M Bosetti C Garavello W Bertuccio P Levi F. Thyroid cancer mortality and incidence: a global overview. Int J Cancer. (2015) 136:2187–95. 10.1002/ijc.29251 25284703

[29] Ahn HS Kim HJ Kim KH Lee YS Han SJ Kim Y Thyroid cancer screening in South Korea increases detection of papillary cancers with no impact on other subtypes or thyroid cancer mortality. Thyroid. (2016) 26:1535–40. 10.1089/thy.2016.0075 27627550

[30] Li M Brito JP Vaccarella S Salvatore V. Long-term declines of thyroid cancer mortality: an international age-period-cohort analysis. Thyroid. (2020) 30:838–46. 10.1089/thy.2019.0684 31964280

